# Clinical Application of Inhaled Nitric Oxide in Conditions of Excessive Right Heart Load: A Review from Neonatal Pulmonary Hypertension to Perioperative Cardiac Surgery Management

**DOI:** 10.3390/jcdd13020081

**Published:** 2026-02-08

**Authors:** Chengming Hu, Zhe Chen, Lizhi Lv, Yan Zhu, Yan Chen, Qiang Wang

**Affiliations:** 1 Department of Pediatric Cardiac Surgery, Beijing Anzhen Hospital, Capital Medical University, No. 2 Anzhen Street, Chaoyang District, Beijing 100029, China; happyhcm@mail.ccmu.edu.cn (C.H.); chenzhe3772@vip.163.com (Z.C.); lvlihi@mail.ccmu.edu.cn (L.L.); 2 Department of ICU in Pediatric Cardiology, Beijing Anzhen Hospital, Capital Medical University, No. 2 Anzhen Street, Chaoyang District, Beijing 100029, China; 18810797747@163.com (Y.Z.); 13501364617@163.com (Y.C.)

**Keywords:** inhaled nitric oxide, excessive right heart load, pulmonary hypertension, perioperative management

## Abstract

Excessive right heart load imposes an acute or chronic injury on the right ventricle (RV), predisposing critically ill neonates and cardiac surgical patients to RV failure, low cardiac output syndrome, and death. Inhaled nitric oxide (iNO) is a selective pulmonary vasodilator that improves ventilation–perfusion matching and unloads the RV without systemic hypotension; nonetheless, its application beyond established neonatal indications remains contentious. Our review synthesizes current mechanistic, translational, and clinical evidence regarding iNO use in three major settings characterized by excessive RV load: (1) neonatal pulmonary hypertension, particularly PPHN; (2) acute and chronic RV overload in older children and adults, including secondary pulmonary hypertension, acute respiratory distress syndrome (ARDS), and acute pulmonary embolism; and (3) perioperative and post-cardiopulmonary bypass (CPB) management in congenital and adult cardiac surgery. In term and near-term infants with hypoxic respiratory failure, pivotal randomized trials show that iNO consistently improves oxygenation and reduces extracorporeal membrane oxygenation (ECMO) use, but this has little effect on survival and long-term neurodevelopment. In ARDS and other adult critical-care indications, iNO provides transient improvements in gas exchange and RV performance without reducing mortality or ventilator duration, and meta-analyses signal an increased risk of acute kidney injury, particularly with prolonged use. In contrast, perioperative studies around CPB demonstrate that prophylactic postoperative iNO and intra-CPB nitric oxide administration can attenuate pulmonary hypertensive crises, facilitate separation from CPB, shorten ventilation and intensive care stay, and, in selected high-risk cohorts, may reduce cardiac surgery-associated acute kidney injury, although survival benefits remain unproven. Across these scenarios, iNO should be used judiciously and in a pathophysiology-driven manner as a time-limited, targeted adjunct to stabilize patients with documented or anticipated RV strain rather than a disease-modifying therapy. Future work should refine patient selection, timing, dosing, and weaning strategies, and define the long-term safety and cost-effectiveness of iNO within contemporary multimodal RV support pathways.

## 1. Introduction

### 1.1. Background

Nitric oxide (NO) is a gaseous signaling molecule synthesized endogenously by nitric oxide synthases (NOS) in vascular endothelium, where it plays an important role in the regulation of vascular tone, platelet function, and inflammation [1]. Across both the systemic and pulmonary vasculatures, endothelial-derived NO diffuses into adjacent vascular smooth muscle cells, activates soluble guanylate cyclase, and increases cyclic guanosine monophosphate (cGMP), thereby promoting smooth-muscle relaxation and vasodilation [2]. Pharmacologic delivery of NO by inhalation (inhaled nitric oxide, iNO) exploits these same pathways but confines the vasodilatory effect to the pulmonary vasculature: NO is delivered to ventilated alveoli, diffuses into the pulmonary capillary bed, and is rapidly bound and inactivated by hemoglobin, thereby avoiding the systemic vasodilation and hypotension induced by intravenous vasodilators [3].

An increase in pulmonary vascular resistance (PVR), whether acute or chronic, imposes an excessive pressure load on the right ventricle (RV) [4,5,6]. In the acute setting, a steep rise in PVR abruptly increases RV afterload, reduces RV stroke volume, and can lead to RV dilation, an interventricular septal shift, and impaired left ventricular filling, ultimately lowering systemic cardiac output and hypotension [4,5,7]. Chronic elevation of PVR drives RV hypertrophy, progressive dilatation, and eventual RV failure [4,5]. These hemodynamic disturbances are particularly hazardous in perioperative and critical care settings, where small deteriorations in RV function may precipitate circulatory collapse. A selective pulmonary vasodilator that can rapidly reduce PVR without compromising systemic arterial pressure is, therefore, an attractive therapeutic option in patients with an already vulnerable RV [8,9,10].

### 1.2. Disease Burden and Clinical Impact

In clinical practice, many diseases can lead to an increase in pulmonary vascular resistance [11,12]. Persistent Pulmonary Hypertension of the Newborn (PPHN) is a rare but extremely threatening condition that can cause hypoxic respiratory failure in full-term and near-term infants. Numerous instances necessitate mechanical breathing and high-concentration oxygen treatment. When pharmacological intervention proves futile, the implementation of extracorporeal membrane oxygenation (ECMO) technology is necessary. These survivors frequently experience prolonged NICU stays and an increased risk of long-term neurodevelopmental, auditory, and respiratory sequelae [11,13,14]. Complex congenital heart disease (CHD) is another major source of morbidity related to excessive right ventricular (RV) load. Lesions with increased pulmonary blood flow or pre-existing pulmonary hypertension often require early repair under cardiopulmonary bypass (CPB). In this setting, the reactive pulmonary vasculature and elevated PVR predispose patients to perioperative pulmonary hypertensive crises, RV failure, and low cardiac output syndrome (LCOS). These complications are associated with higher perioperative mortality, increased inotrope and ECMO use, and prolonged ICU and hospital stays [15,16,17]. Similar hemodynamic challenges are seen in adults with secondary pulmonary hypertension due to left heart disease, chronic lung disease, or acute respiratory distress syndrome (ARDS), particularly during and after CPB [12]. Across these settings, current therapies—systemic vasodilators and inodilators—can reduce PVR but frequently cause systemic hypotension and impaired organ perfusion, while ECMO, although effective, is invasive, resource-intensive, and prone to complications [18,19].

### 1.3. Existing Therapeutic Strategies and Limitations

Currently, management of excessive PVR and RV load relies on a combination of high inspired oxygen, optimized ventilatory strategies (including high-frequency ventilation), inotropic and inodilator support (e.g., dobutamine, milrinone), and vasodilators with both systemic and pulmonary effects such as prostacyclin analogs or phosphodiesterase-5 inhibitors [20,21]. In refractory cases, ECMO is used as a salvage option to support gas exchange and circulation while PVR gradually falls or the underlying disease is treated [19,22,23].

These approaches are limited by non-selective vasodilation, systemic hypotension, and the need for high-resource infrastructure, particularly for prostacyclin infusions and ECMO. Such resources are often unavailable in resource-limited settings [24,25]. In this context, iNO is conceptually attractive as a selective pulmonary vasodilator and RV afterload reducer: it is delivered to ventilated lung units and rapidly inactivated in the bloodstream. It can lower PVR and improve ventilation–perfusion matching with minimal impact on systemic arterial pressure, filling an important therapeutic gap in the management of patients with excessive right heart load [18,26,27,28].

### 1.4. Objectives and Scope of the Review

The aim of this review is to summarize the clinical application of iNO in three key settings characterized by excessive right ventricular load: (1) neonatal pulmonary hypertension, particularly persistent pulmonary hypertension of the newborn (PPHN); (2) diseases with excessive right heart load in children and adults, including secondary pulmonary hypertension and acute or chronic right ventricular failure; and (3) patients undergoing cardiopulmonary bypass, with a focus on intraoperative and early postoperative periods. Our review updates the perioperative iNO evidence base with recent RCTs and large post-marketing studies, and integrates them into practical algorithms for right-heart-focused perioperative care. Because several studies span more than one clinical scenario (e.g., mixed neonatal, pediatric and perioperative contexts), we assigned each study to a primary category based on the predominant population and timing of iNO use; the classification is presented in Appendix A Table A1.

**Figure 1:**
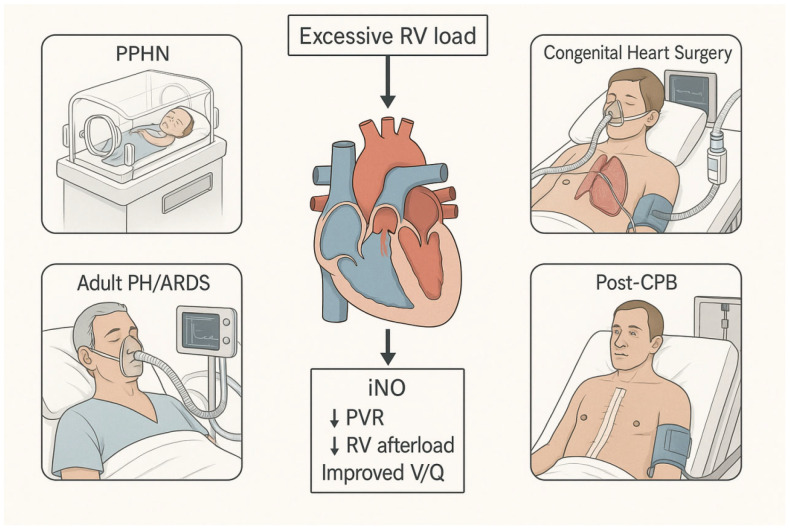
Clinical Scenarios of Excessive Right Ventricular Load and the Therapeutic Role of Inhaled Nitric Oxide.

We emphasize clinical evidence from randomized and observational studies, integrate relevant pathophysiologic rationale, and critically examine current controversies regarding efficacy, safety, dosing, and cost-effectiveness. Particular attention will be paid to the surgical and perioperative implications of iNO use in congenital and adult cardiac surgery. Table 1 and Figure 1 will schematically depict the major clinical scenarios associated with excessive right ventricular load (PPHN, congenital heart surgery, adult PH/ARDS, and post-CPB RV dysfunction) and illustrate where iNO is typically incorporated into management algorithms. Other rare and heterogeneous entities (e.g., pulmonary vein obstruction and complex congenital shunt physiology, such as a large PDA or d-TGA with PH) are beyond the scope of this review and typically require individualized hemodynamic assessment [29,30].

## 2. Theoretical Background

### 2.1. Key Phenotype-Specific Mechanisms Across PH Groups

The predominant cause of PH phenotypes, and consequently the anticipated function of iNO, varies by WHO classification and clinical scenario (Table 2) [30]. Neonatal PH, PPHN and perioperative (e.g., CPB-related) reactive PH are characterized mainly by acute, recruitable vasoconstriction and endothelial dysfunction with reduced NO bioavailability, making iNO most plausibly effective for rapid, lung-selective PVR reduction and RV unloading [47]. Group 1 PAH combines endothelial imbalance with progressive arteriolar remodeling, so iNO may be useful for short-term stabilization or vasoreactivity assessment, but responses diminish as fixed structural disease predominates. Group 2 PH is primarily post-capillary with elevated left-sided filling pressures, with some patients developing combined post- and pre-capillary remodeling; in this setting, selective pulmonary vasodilation may increase pulmonary blood flow and worsen pulmonary edema, so iNO should be a tightly monitored in a short trial with clear stopping rules. Group 3 PH associated with lung disease reflects hypoxic vasoconstriction, inflammatory endothelial injury, microthrombosis, and marked V/Q heterogeneity; iNO can transiently improve oxygenation via V/Q redistribution and may reduce RV afterload when ventilated units are recruitable, but it does not reverse underlying parenchymal pathology. Group 4 PH is driven by pulmonary arterial obstruction, where iNO serves mainly as short-term RV support rather than definitive therapy. Group 5 PH is multifactorial and heterogeneous, often presenting mixed pre-/post-capillary components, leading to unpredictable iNO responsiveness and emphasizing individualized, mechanism-guided use.

### 2.2. Pathophysiology of Excess Right Heart Load

The right ventricle (RV) is coupled to low-pressure, high-compliance pulmonary circulation, so even modest acute or chronic rises in PVR markedly increase RV afterload, leading to RV dilation, septal shift, reduced left ventricular filling, and ultimately systemic hypotension and organ hypoperfusion [48,49]. In neonates, failure of the normal postnatal decline in PVR maintains a fetal pattern of circulation with persistent patency of the ductus arteriosus and foramen ovale, resulting in right-to-left shunting, severe hypoxemia, and progressive RV dysfunction [50]. After cardiopulmonary bypass (CPB), blood–surface interactions trigger contact activation, referring to activation of the intrinsic coagulation and contact system (factor XII–kallikrein–kinin), with downstream complement, inflammatory cascades and platelet activation [51,52]; together with ischaemia–reperfusion injury, and inflammatory mediator release, these processes promote endothelial dysfunction and vasoconstriction in the pulmonary bed, acutely raising PVR and predisposing susceptible patients to RV failure and pulmonary hypertensive crises [53,54,55]. In adult patients, when ARDS is accompanied by secondary pulmonary hypertension, acute pulmonary embolism, and decompensated chronic pulmonary hypertension, the right ventricle is also subjected to similar hemodynamic pressure effects. In these cases, acute or progressive obstruction and remodeling of the pulmonary vessels suddenly increase the afterload of the right ventricle and may lead to rapid hemodynamic collapse [12].

### 2.3. Endogenous NO and Exogenous iNO

#### 2.3.1. Endogenous NO Production and the NO–sGC–cGMP Axis

Endogenous NO is generated from L-arginine by nitric oxide synthase (NOS) isoforms, with endothelial NOS (eNOS) being the dominant vascular source [2]. Shear stress, oxygen tension, and cofactors such as tetrahydrobiopterin regulate eNOS coupling; oxidative stress can uncouple eNOS and reduce NO bioavailability. NO diffuses into pulmonary arterial smooth-muscle cells and activates soluble guanylate cyclase (sGC), increasing intracellular cyclic guanosine monophosphate (cGMP) [56]. cGMP activates protein kinase G, decreases intracellular calcium, and promotes smooth-muscle relaxation; it also exerts anti-proliferative and anti-platelet effects that are relevant to chronic pulmonary vascular disease [57].

cGMP signaling is terminated primarily by phosphodiesterase enzymes, especially phosphodiesterase type 5 (PDE5), which is highly expressed in the pulmonary vasculature and upregulated in several PH phenotypes [58]. PDE5 inhibitors (e.g., sildenafil and tadalafil) can potentiate NO-mediated vasodilation and help prevent rebound PH during iNO weaning. Milrinone (a PDE3 inhibitor) may be used as adjunct support, but because it dilates both systemic and pulmonary arteries, it can cause systemic hypotension and should be carefully titrated with hemodynamic monitoring [59]. Conversely, in patients with elevated left-sided filling pressures, augmenting pulmonary blood flow through NO–cGMP pathways may increase pulmonary edema risk; thus, combined use requires careful titration and hemodynamic monitoring.

#### 2.3.2. Exogenous iNO

Clinically, iNO is delivered via the inspiratory limb of the ventilatory circuit or through nasal continuous positive airway pressure and high-flow systems in spontaneously breathing patients, allowing precise titration of the inspired concentration [60,61]. Inhaled nitric oxide (iNO) induces selective pulmonary vasodilation via administered directly to ventilated alveoli, diffusion into neighboring pulmonary vessels, and swift binding and inactivation by hemoglobin, thereby confining its effects to pulmonary circulation and reducing systemic vasodilation [18,26,27]. However, iNO responsiveness depends on the presence of recruitable, well-ventilated lung units. In patients with extensive consolidation, atelectasis and alveolar flooding (e.g., surfactant deficiency, edema, and pneumonia), iNO delivery to functional alveoli may be limited, so it is necessary to first optimize the expansion effect of the lungs and treat any potential lung parenchymal lesions before iNO [37]. In clinical practice, iNO is generally administered at concentrations ranging from 5 to 40 parts per million (ppm), exhibiting a swift onset and offset of effect within minutes of dose modification, showing that most oxygenation and hemodynamic benefit is achieved at relatively low doses (20 ppm in many neonatal and adult studies) [8,31,32]. It also demonstrates a distinct “ceiling effect,” where further increases in dosage beyond a modest threshold (typically 20–40 ppm) yield minimal additional enhancement in oxygenation or hemodynamics [31,32]. Upon absorption, NO interacts with haemoglobin to produce methaemoglobin (metHb) and nitrate; it also interacts with thiol-containing compounds to yield nitrite and S-nitrosothiols, which may serve as a circulating reservoir of NO and contribute to more nuanced systemic effects [9,62,63].

### 2.4. Preclinical and Translational Evidence

Recent experimental work has refined our understanding of how NO-based interventions interact with pulmonary vascular mechanics and remodeling. In a monocrotaline-induced PAH rat model, inhaled NO produces frequency-dependent improvements in pulmonary arterial impedance, suggesting that the mechanical response to NO varies with disease state rather than reflecting a uniform vasodilatory effect [64]. In hypoxia-induced PH models, modulation of multiple nodes along the NO–sGC–cGMP pathway (e.g., augmenting NO availability and limiting cGMP degradation) attenuated pulmonary vascular changes and right ventricular remodeling, supporting the concept that impaired NO signaling is both a marker and a mechanistic contributor to experimental PH progression [65]. These in vivo findings complement clinical observations by highlighting when NO responsiveness may reflect recruitable vasoconstriction versus fixed structural disease.

Mechanistic insight from in vitro and cellular systems further supports phenotype-specific NO biology. In the Sugen–hypoxia model, carbonic anhydrase inhibition improved pulmonary artery reactivity and enhanced NO-mediated relaxation mechanisms, illustrating that restoring vasodilatory reserve can be model- and pathway-dependent [66]. Cellular studies also implicate dysregulated NOS activity in remodeling: macrophage inducible NOS (iNOS) signaling has been linked to proliferative crosstalk with adjacent pulmonary artery smooth muscle cells, providing a plausible inflammatory–NO axis for vascular remodeling [67]. In ARDS, iNOS-driven high-output NO together with inflammation-associated superoxide favors rapid peroxynitrite (ONOO−) formation (k = 1.6 × 10^−10^ M^−1^s^−1^), which can contribute to nitro-oxidative tissue injury and may help explain the limited response to iNO [68,69,70]. Finally, translational formulation work has tested alternative NO-delivery strategies in small-animal lung injury models; for example, NO-releasing nanoparticles reduced inflammatory injury in an LPS-induced ARDS model, supporting ongoing development of NO-based platforms beyond conventional gaseous delivery [71]. Collectively, these experimental studies provide updated mechanistic rationale for future research, including phenotype-guided use and combination strategies targeting the NO–cGMP axis.

### 2.5. Clinical Delivery, Monitoring and Safety

Inhaled nitric oxide is usually supplied as a compressed gas and delivered via a calibrated injector/blender with inline monitoring of NO, NO_2_ and FiO_2_ integrated into the ventilator circuit [72,73,74]. It can be administered through conventional invasive mechanical ventilation, high-frequency oscillatory ventilation, selected non-invasive or high-flow systems, and, in the cardiac surgical setting, by adding NO to the sweep gas of the CPB oxygenator [75,76]. Safe use requires regular measurement of methaemoglobin, continuous surveillance of delivered NO and NO_2_ concentrations with alarm thresholds, adherence to recommended exposure limits for staff, and appropriate scavenging to minimize ambient contamination [35,77,78]. Recognized adverse effects include dose-related methaemoglobinemia, NO_2_-induced pulmonary toxicity at excessive concentrations, rebound pulmonary hypertension with abrupt withdrawal, and potential oxidative or nitrosative stress with prolonged or high-dose therapy; key delivery modes, target doses, monitoring parameters, and safety thresholds are summarized in Table 3.

## 3. Research Status

### 3.1. Neonatal Pulmonary Hypertension and Hypoxic Respiratory Failure

The pivotal randomized trials of iNO in term and near-term infants with hypoxic respiratory failure involved enrolling mechanically ventilated neonates ≥ 34 weeks of gestation with oxygenation index (OI) ≥ 25 (OI = FiO_2_ × MAP × 100/PaO_2_) and no structural heart disease, and comparing conventional therapy alone with adjunctive iNO [31,79]. In the multicenter Neonatal Inhaled Nitric Oxide Study Group trial, infants received 20 ppm iNO (with escalation to 80 ppm in non-responders) or 100% oxygen, and iNO significantly reduced the composite of death or ECMO at 120 days (46% vs. 64%) by lowering ECMO use (39% vs. 54%), while mortality was unchanged (14% vs. 17%) [31]. In the low-dose trial by Clark et al., neonates meeting similar criteria received 20 ppm iNO for up to 24 h followed by 5 ppm for ≤96 h versus control gas, leading to a reduction in ECMO requirement from 64% to 38%, with no difference in 30-day mortality (7% vs. 8%) and a lower incidence of chronic lung disease (7% vs. 20%) [32]. Collectively, these RCTs demonstrate that iNO reliably improves oxygenation and decreases the need for ECMO in severe neonatal pulmonary hypertension, but confers little or no survival advantage.

### 3.2. Diseases with Excessive Right Heart Load in Children and Adults

#### 3.2.1. ARDS with Secondary Pulmonary Hypertension

In adults and children with ARDS and secondary pulmonary hypertension, randomized trials and meta-analyses show that iNO produces only transient physiological benefit without improving survival [8,80,81,82]. More recently, high-dose iNO has been explored in COVID-19-related acute hypoxemic respiratory failure; a multicenter phase II trial reported improved oxygenation at 48 h versus usual care, but was not powered to detect differences in mortality or longer-term outcomes [83]. The Cochrane review (14 RCTs, ~1300 patients) found a significant increase in PaO_2_/FiO_2_ at 24 h (mean difference 15.9, 95% CI 8.3–23.6) and an improved oxygenation index, but no reduction in longest-follow-up mortality (38.2% vs. 37.5%; RR 1.04, 95% CI 0.90–1.19), no gain in ventilator-free days, duration of mechanical ventilation, or ICU/hospital length of stay [80]. A separate meta-analysis of nine ARDS trials (*n* = 1142) likewise showed no mortality benefit in either severe (PaO_2_/FiO_2_ ≤ 100; RR 1.01, 95% CI 0.78–1.32) or mild–moderate ARDS (RR 1.12, 95% CI 0.89–1.42) [84]. Importantly, iNO was associated with an increased risk of renal impairment (RR 1.59, 95% CI 1.17–2.16) [80]. These data support the physiological theoretical basis—in ventilated lung units, selective pulmonary vascular dilation can improve the matching of ventilation and perfusion, reduce pulmonary vascular resistance, and quickly alleviate the burden on the right ventricle. But at the same time, they also indicate that this cannot translate into better long-term efficacy. Therefore, in acute ARDS, iNO should be regarded as a short-term emergency treatment for refractory hypoxemia and right ventricular dysfunction, rather than a treatment that can improve the condition, particularly considering its potential adverse effects, substantial cost and resource implications [8,80,81].

#### 3.2.2. Acute Pulmonary Embolism and Acute RV Failure

In acute pulmonary embolism (PE) with right ventricular (RV) failure, evidence for iNO comes mainly from studies with small case series and one randomized trial [85,86,87,88,89]. Case series of massive PE treated with 20–25 ppm iNO report rapid improvements in pulmonary and systemic arterial pressures, heart rate, and gas exchange within minutes, with all four patients in one series surviving to hospital discharge [85,86]. A systematic review of iNO for acute PE identified a majority of case reports and small cohorts (plus an 8-patient phase I trial) and concluded that most patients showed prompt gains in oxygenation and hemodynamics; however, the data were insufficient to prove safety or efficacy or any mortality benefit [87,88]. In the multicenter iNOPE randomized trial of 76 patients with submassive PE and RV dysfunction, 50 ppm iNO for 24 h did not significantly increase the proportion of patients achieving a composite endpoint of normal RV size/function and low troponin (13% placebo vs. 24% iNO; *p* = 0.375), although a pre-planned post hoc analysis showed a higher rate of resolution for RV hypokinesis/dilation with iNO (+29%; *p* = 0.010) [89]. Beyond PE, observational data in cardiogenic shock and severe RV failure (often on VA-ECMO or ECPELLA) suggest that iNO can improve RV performance and device flows and may facilitate earlier weaning from mechanical support, but without robust controlled outcome data [90,91]. Overall, these studies support the use of iNO as a rescue bridge therapy to acutely unload the RV and stabilize oxygenation while definitive treatment (anticoagulation, thrombolysis, catheter/surgical embolectomy, or mechanical circulatory support) is instituted, rather than as a stand-alone, outcome-modifying therapy for acute PE or RV failure [87,89,91].

In adult diseases characterized by excessive load on the right ventricle, iNO can reliably provide short-term physiological benefits, enabling rapid improvement in oxygenation, pulmonary artery pressure, and right ventricular function in many patients. However, in this study, iNO did not significantly reduce mortality, mechanical ventilation time, or length of stay in the intensive care unit, and long-term use may cause damage to the kidneys. Taken together, these data support positioning iNO not as a chronic or disease-modifying therapy, but as a targeted, time-limited support tool—used to transiently reduce RV afterload and stabilize gas exchange while definitive treatments (reperfusion, surgery, optimization of heart failure and PH therapy, or mechanical support) are implemented.

### 3.3. Perioperative and Post-Cardiopulmonary Bypass Use

#### 3.3.1. Rationale in Cardiac Surgery

Cardiopulmonary bypass (CPB) triggers a systemic inflammatory and ischaemia–reperfusion response that promotes endothelial dysfunction, oxidative stress and vasoconstriction in the pulmonary circulation, leading to increased PVR, impaired RV–pulmonary artery coupling and a predictable fall in cardiac output after surgery; in infants and children with pre-existing high pulmonary flow or pressure, this milieu predisposes to postoperative pulmonary hypertensive crises, RV failure and LCOS [53,54,92,93]. iNO acting directly on pulmonary vascular smooth muscle and rapidly inactivated by hemoglobin, can counteract this surge in PVR, improve RV forward flow and attenuate the cascade leading to PHT crises and LCOS, providing a strong mechanistic rationale for its prophylactic or early postoperative use in high-risk congenital and adult cardiac surgery [15,38,54].

#### 3.3.2. Prophylactic Postoperative iNO in Congenital Heart Surgery

In congenital heart surgery, the best evidence for prophylactic postoperative iNO comes from the randomized double-blind trial by Miller et al., which enrolled 124 infants (median age 3 months) with high pulmonary flow and/or pressure undergoing corrective surgery for lesions such as large VSD, complete AVSD, truncus arteriosus, or total anomalous pulmonary venous drainage, all with preoperative pulmonary hypertension. Infants were randomized to continuous 10 ppm iNO or placebo from arrival in the ICU until just before extubation [15]. Compared with placebo, iNO significantly reduced pulmonary hypertensive crises (median 4 [IQR 0–12] vs. 7 [1–19] episodes; adjusted RR 0.65, *p* = 0.045) and shortened the time to meeting extubation criteria (80 [38–121] vs. 112 h [63–164], *p* = 0.019), with fewer patients still ventilated at 7 days (10% vs. 26%, *p* = 0.02) and a shorter total time on gas (87 [43–125] vs. 117 h [67–168], *p* = 0.023). Trends toward reduced total ventilation time (117 vs. 140 h) and ICU stay (138 vs. 162 h) favored iNO but did not reach statistical significance; in addition, mortality (6.5% overall) was similar between groups [15]. Collectively, these data support routine low-dose postoperative iNO in selected high-risk congenital repairs to prevent pulmonary hypertensive crises and accelerate postoperative stabilization, while its effects on ICU length of stay and survival remain modest and uncertain [15,16].

#### 3.3.3. Intra-CPB NO Administration

Intraoperative delivery of nitric oxide via the CPB oxygenator has been evaluated most rigorously in the single-center RCT by James et al., in which 198 children undergoing congenital heart surgery were randomized to receive 20 ppm NO blended into oxygenator sweep gas for the entire duration of CPB or standard bypass without NO [17]. Children in the NO group developed LCOS significantly less often than controls (15% vs. 31%, *p* = 0.007), with the greatest benefit observed in those <6 weeks of age (20% vs. 52%, *p* = 0.012) and in those 6 weeks–2 years (6% vs. 24%, *p* = 0.026); in these younger cohorts, ICU length of stay was also reduced (median 43 vs. 84 h, *p* = 0.031). The incidence of LCOS after more complex procedures was similarly lower with intra-CPB NO (17% vs. 48%, *p* = 0.018); ECMO use was also markedly reduced (1% vs. 8%, *p* = 0.014) [17]. Earlier small pediatric and adult studies introducing NO into the CPB circuit demonstrated reductions in biochemical markers of myocardial injury and inflammation and suggested the potential for shorter ventilation and ICU stays, but were underpowered for hard clinical endpoints [94,95,96]. A recent systematic review and meta-analysis pooling pediatric and adult CPB studies similarly suggests improved early postoperative outcomes (e.g., shorter ICU stay), but heterogeneity and limited long-term endpoints preclude firm conclusions [97]. The NITRIC double-blind, multicenter randomized clinical trial (JAMA, 2022; *n* = 1371 children < 2 years) reported that NO delivered into the CPB oxygenator did not increase ventilator-free days compared with standard care, suggesting limited benefit of routine intra-CPB NO supplementation in this population [98]. Taken together, the current evidence does not support routine intra-CPB NO administration. While some single-center trials suggest potential benefits, the large multicenter NITRIC trial failed to show efficacy, indicating that intra-CPB NO should be considered investigational and further studies should be conducted in carefully designed, phenotype- and endpoint-driven trials.

#### 3.3.4. After-CPB NO Administration

After CPB, many congenital and adult cardiac surgery patients develop acute pulmonary hypertension and RV dysfunction that can delay separation from bypass and compromise early postoperative stability [16,38,99]. In infants and children with preoperative pulmonary hypertension who develop elevated pulmonary pressures immediately after CPB, Russell et al. reported that iNO reduced mPAP without systemic hypotension, supporting its role in stabilizing hemodynamics in the early post-bypass period [99]. Evidence for routine prophylactic low-dose postoperative iNO after high-risk congenital heart surgery is discussed in detail in Section 3.3.2 [15]. In adults with severe postoperative pulmonary hypertension, iNO has been shown to lower mPAP more effectively with less systemic hypotension than intravenous prostacyclin [100,101,102], and to maintain higher RV ejection fraction and lower vasopressor requirements compared with milrinone in cardiac surgery patients [102]. Together, these data support inhaled NO as a selective pulmonary vasodilator that facilitates separation from CPB in patients with elevated PVR, stabilizes RV function, and promotes earlier postoperative recovery, particularly in high-risk congenital repairs and adult operations complicated by pulmonary hypertension [15,99,100,101,102]. Across these diverse clinical scenarios, multiple randomized trials and meta-analyses have evaluated the efficacy, safety, and organ-specific effects of iNO. The key randomized controlled trials and systematic reviews with major indications are summarized in Table A1.

## 4. Research Challenges and Controversies

Despite robust physiologic effects on oxygenation and pulmonary artery pressure, most randomized and observational studies of iNO have failed to demonstrate consistent improvements in hard outcomes such as mortality, neurologic sequelae, or long-term functional status. This might be due to the late initiation, short exposure, and the fundamentally multifactorial nature of neonatal PPHN, ARDS, and perioperative low-cardiac output [31,80]. Important uncertainties remain regarding optimal timing, dose, and duration—for example, prophylactic versus rescue use in cardiac surgery, early versus late initiation in PPHN, and the wide heterogeneity in clinical practice (with starting doses of 5–20 ppm or higher, variable titration and weaning schemes) in the absence of robust dose–response trials [38,103]. A rapid cessation of iNO may precipitate a clinically substantial rebound in pulmonary hypertension. Consequently, the dosage must be incrementally diminished, comprehensive hemodynamic monitoring should be performed throughout the drug cessation process, and for high-risk patients, it is essential to administer oral or intravenous pulmonary vasodilators concurrently [104,105]. Safety issues, while normally manageable, encompass methemoglobinemia, pulmonary toxicity associated with nitrogen dioxide, fluid-sensitive pulmonary edema in the presence of left-sided lesions, and the possibility of systemic oxidative or nitrosative stress. The enduring consequences of neonatal exposure on neurodevelopment and pulmonary growth, together with the effects of repeated treatments in geriatric patients, remain inadequately studied [31]. In parallel, the high cost and logistical complexity of iNO delivery (proprietary gas systems, continuous monitoring, and trained staff) in contrast to cheaper systemic alternatives such as sildenafil or prostacyclin, which themselves carry risks of systemic hypotension, and with the extreme resource demands of ECMO, create significant access and health-economic challenges, especially in low- and middle-income settings [35,106,107]. Finally, much of the evidence base is limited by small, single-center trials, heterogeneous patient populations and outcome definitions, and short follow-up periods focused on physiological or ICU end points, particularly in CPB-related studies and adult right-heart failure, leaving key questions about long-term efficacy, safety, and cost-effectiveness unanswered [38,103].

## 5. Conclusions and Future Directions

Inhaled nitric oxide now has an established role as a standard of care in term and near-term infants with persistent pulmonary hypertension of the newborn and severe hypoxic respiratory failure, where it reliably improves oxygenation and reduces ECMO use. In older children and adults, it functions mainly as an adjunctive therapy—a selective, short-acting pulmonary vasodilator used perioperatively in cardiac surgery for pulmonary hypertension and vulnerable right ventricles, and as temporary support in acute RV failure (e.g., ARDS-related PH, acute PE, post-CPB PH crises). In these settings, iNO is best viewed as a targeted tool to acutely reduce PVR, unload the RV, and stabilize gas exchange rather than a disease-modifying or chronic therapy, and should be integrated into multimodal RV support alongside inotropes, vasopressors, other pulmonary vasodilators, and, when necessary, mechanical circulatory support [8,80]. For surgeons, anesthesiologists, and intensivists, the practical take-home message is that iNO should be implemented judiciously and phenotype-specifically, with a clear understanding of the underlying pathophysiology and patient-specific hemodynamics, and delivered through standardized protocols for initiation, monitored response, titration, and weaning within a multidisciplinary team.

Looking forward, key research priorities include adequately powered multicenter RCTs in clearly defined high-risk subgroups (such as complex congenital heart surgery and adult RV failure), dose-finding and timing studies—including intra-CPB vs. postoperative strategies—systematic long-term follow-up of neonatal and pediatric cohorts, and robust cost-effectiveness and implementation analyses across different health systems. Mechanistic work on myocardial and distant organ protection, and on systemic NO biology in the context of CPB and critical illness, will help refine when and how iNO is most beneficial. Taken together, the existing evidence and strong physiologic rationale support a continued but judicious use of iNO: when applied in the right patient, at the right time, and embedded in comprehensive perioperative and critical-care pathways, it remains a valuable tool for managing conditions of excessive right heart load.

## Figures and Tables

**Table 1:** Clinical scenarios with excessive right ventricular (RV) load and the role of inhaled nitric oxide (iNO).

Clinical Scenario	Mechanism of Excessive RV Load/↑ PVR	Typical Clinical Consequences	Role of Inhaled NO (iNO)	References
Persistent pulmonary hypertension of the newborn (PPHN)	Failure of normal postnatal fall in PVR; sustained fetal circulatory shunts	Severe hypoxemia, RV dysfunction, need for ECMO, NICU stay	Selective pulmonary vasodilator to ↓ PVR, improve oxygenation, reduce ECMO requirement	[31,32,33,34]
Neonatal hypoxic respiratory failure (meconium, sepsis, RDS)	Hypoxia, acidosis, and lung injury causing reactive pulmonary vasoconstriction	Refractory hypoxemia, ventilator dependence, hemodynamic lability	Rescue therapy to improve V/Q matching and unload RV in selected responders; benefit may be limited with consolidation/atelectasis/flooding	[31,32,33,35,36,37]
Complex congenital heart disease requiring CPB (pediatric)	Pre-existing or reactive pulmonary hypertension; CPB-induced endothelial dysfunction	Pulmonary hypertensive crises, LCOS, RV failure, prolonged ICU stay	Prophylactic or rescue use to prevent/treat PHT crises and facilitate postoperative stabilization	[15,38,39]
Adult pulmonary hypertension/ARDS with secondary PH	Hypoxic vasoconstriction, vascular remodeling, microthrombi in pulmonary circulation	RV dilatation, reduced RV output, refractory hypoxemia	Short-term support to ↓ PVR, improve oxygenation and RV performance (no proven mortality benefit)	[28,40,41,42]
Post-CPB RV dysfunction (adult cardiac surgery)	CPB-related inflammation, ischaemia–reperfusion, increased PVR and RV afterload	Difficulty weaning from CPB, LCOS, need for high inotrope/ECMO support	Adjunct to facilitate separation from CPB, stabilize RV, and reduce need for aggressive systemic vasodilators	[38,43,44]
Acute RV failure from other causes (e.g., acute PE, decompensated PH)	Abrupt rise in PVR (embolus, crisis in chronic PH)	Acute RV failure, systemic hypotension, cardiogenic shock	Bridge therapy to reduce RV afterload and improve hemodynamics while definitive treatment is instituted	[45,46]
General principle across scenarios	↑ PVR → ↑ RV afterload → RV dilatation/dysfunction → ↓ LV filling and output	Systemic hypotension, end-organ hypoperfusion, high mortality	iNO acts as aselective pulmonary vasodilator and RV afterload reducer with minimal systemic hypotension	[4,5,28,40,41]

**Table 2:** WHO PH groups: core mechanisms and iNO role.

PH Phenotype	Core Pathophysiology	Implications for iNO
Group 1 PAH (vascular remodeling of pulmonary arteries)	Endothelial dysfunction (↓ NO/PGI2, ↑ endothelin) + vascular remodeling; variable vasoreactivity	Variable response; useful for acute RV unloading, perioperative crises, or vasoreactivity testing; limited in fixed remodeling
Group 2 PH associated with left-sided heart disease	Post-capillary PH from high LA pressure; may progress to combined post/pre-capillary disease	Often limited; can worsen pulmonary edema if LV filling leads to high pressures—use only with careful echo/hemodynamic assessment
Group 3 PH associated with lung disease	Hypoxic vasoconstriction + inflammatory endothelial injury; microthrombi; V/Q heterogeneity; mechanical compression	Transient V/Q improvement and RV unloading in ventilated units; no mortality benefit; avoid prolonged use (AKI signal)
Group 4 PH associated with pulmonary artery obstructions	Acute: obstruction + mediator-driven vasoconstriction; Chronic: fixed obstruction/remodeling	Most plausible as short-term bridge in acute PE with RV dysfunction while reperfusion or anticoagulation proceeds; limited in chronic fixed disease
Group 5 PH with unclear and/or multifactorial mechanisms	Heterogeneous: systemic/inflammatory or granulomatous lung disease (e.g., sarcoidosis), hematologic disorders (e.g., hemolysis), CKD, metabolic disorders, tumor or fibrosing mediastinitis causing vascular or airway compression; often mixed pre-/post-capillary components.	Evidence limited and response unpredictable; consider a short monitored trial only for acute decompensation with suspected reversible vasoconstriction and RV failure. Discontinue if no objective oxygenation or hemodynamic improvement; prioritize treating the underlying cause (inflammation, anemia, hemolysis, obstruction, compression, metabolic drivers).

**Table 3:** Practical aspects of inhaled nitric oxide (iNO) administration.

Aspect	Option/Parameter	Typical Practice/Key Points
Delivery source	Compressed gas cylinder (e.g., 800–1000 ppm NO in N_2_)	Standard hospital supply; requires dedicated regulator, injector, and inline gas monitoring.
	Integrated NO delivery system	Commercial systems combining blender, flow control, and NO/NO_2_/O_2_ monitoring.
Connection to circuit	Invasive mechanical ventilation	NO injected into inspiratory limb close to ventilator; continuous NO/NO_2_/FiO_2_ monitoring required.
	High-frequency ventilation (HFOV/HFJV)	NO introduced into bias flow; verify stable NO concentration at patient Y-piece.
	Non-invasive/high-flow systems	Possible in selected devices with closed interfaces; risk of gas leakage and ambient exposure must be controlled.
	Cardiopulmonary bypass (CPB) oxygenator sweep gas	NO blended into oxygenator sweep gas during CPB; dosing referenced to sweep gas flow, not minute ventilation.
Typical starting dose	Neonates/children	10–20 ppm (often start at 20 ppm in PPHN/high-risk CHD), then titrate down to lowest effective dose.
	Adults	5–20 ppm in ARDS, acute PH or RV failure; higher doses rarely needed.
	Maximum dose (short term)	Generally ≤40 ppm in most protocols; up to 80 ppm used short-term in some rescue settings with close monitoring.
Titration & weaning	Up-titration	Increase in small steps (e.g., 5–10 ppm) based on oxygenation and PAP/RV response.
	Weaning	Gradual reduction (e.g., 20 → 10 → 5 → 2 → 1 ppm) with clinical and hemodynamic monitoring to avoid rebound PH.
Gas monitoring	NO	Continuous monitoring at patient side; maintain at prescribed setpoint.
	NO_2_	Keep < 2 ppm (many centers aim < 1 ppm); alarms for rapid rise.
	FiO_2_	Continuous monitoring; avoid unnecessary hyperoxia.
Patient monitoring	Methaemoglobin (MetHb)	Check at baseline and regularly thereafter; aim < 5% (many centers intervene at 3–5%).
	Hemodynamics	HR, BP, central venous pressure, PAP/RV function (echo or catheter if available).
	Gas exchange	SpO_2_, arterial blood gases, lactate; assess response within minutes of dose change.
Safety thresholds	NO_2_	Alarm if ≥2 ppm; investigate device setup, scavenging, and FiO_2_.
	MetHb	Reduce iNO dose and/or treat if ≥5%; consider alternative therapies.
	Ambient NO/NO_2_ (staff exposure)	Maintain below occupational limits (e.g., time-weighted thresholds per local regulations); ensure adequate scavenging.
Recognized adverse effects	Methaemoglobinemia	Dose- and duration-related; usually reversible with dose reduction or methylene blue if severe.
	NO_2_ toxicity	Risk of airway/pulmonary injury at high NO_2_; controlled by strict monitoring and alarm limits.
	Rebound pulmonary hypertension	Can occur with abrupt withdrawal; prevent by slow weaning and overlapping oral/IV pulmonary vasodilators if needed.
	Oxidative/nitrosative stress	Theoretical or subtle risk with prolonged/high-dose exposure; minimize dose and duration consistent with goals.
Operational issues	Transport on iNO	Use portable delivery/monitoring units; secure cylinders and ensure battery backup.
	Infection control & maintenance	Regular calibration, filter changes, and device disinfection per manufacturer and hospital protocols.

## Data Availability

All data in this study are available.

## Abbreviations

The following abbreviations are used in this manuscript:

RV	Right ventricle
iNO	Inhaled nitric oxide
ARDS	Acute respiratory distress syndrome
CPB	Cardiopulmonary bypass
ECMO	Extracorporeal membrane oxygenation
NO	Nitric oxide
NOS	Nitric oxide synthases
cGMP	Cyclic guanosine monophosphate
PVR	Pulmonary vascular resistance
PPHN	Persistent pulmonary hypertension of the newborn
CHD	Congenital heart disease
LCOS	Low cardiac output syndrome
PH	Pulmonary hypertension
metHb	methaemoglobin
ICU	Intensive care unit
NICU	Neonatal intensive care unit
mPAP	Mean pulmonary artery pressure
PAP	Pulmonary artery pressure
PAH	Pulmonary arterial hypertension
sGC	Soluble guanylate cyclase
PDE5	Phosphodiesterase type 5
FiO_2_	Fraction of inspired oxygen
MAP	Mean airway pressure
PaO_2_	Partial pressure of arterial oxygen
SpO_2_	Peripheral oxygen saturation
V/Q	Ventilation/perfusion ratio
RCT	Randomized controlled trial
CI	Confidence interval
RR	Risk ratio
IQR	Interquartile range
PE	Pulmonary embolism
AKI	Acute kidney injury
OI	Oxygenation index
HFOV	High-frequency oscillatory ventilation
HFJV	High-frequency jet ventilation
LV	Left ventricle
VSD	Ventricular septal defect
AVSD	Atrioventricular septal defect

## Appendix A

**Table A1:** Key randomized trials and meta-analyses of inhaled nitric oxide (iNO) in major clinical scenarios.

Study/Year	Population & Clinical Setting	N	iNO Dose & Timing	Primary Endpoint	Key Findings
Neonatal Inhaled Nitric Oxide Study Group (NINOS), 1997 [31]	Term/near-term neonates with severe hypoxic respiratory failure (often with PPHN)	235	Rescue iNO, starting at 20 ppm (titrated up to ~80 ppm), continued until oxygenation improved or for ≤96 h	Death or need for ECMO	iNO significantly improved oxygenation and reduced the composite of death or ECMO mainly by lowering ECMO use; no clear effect on mortality alone; no signal for increased renal injury.
Clark et al. (CINRGI), 2000 [32]	≥34-week neonates with PPHN and hypoxic respiratory failure (oxygenation index ≥25)	248	20 ppm for 24 h, then reduced to 5 ppm, continued for up to 96 h as rescue therapy	Need for ECMO	iNO reduced ECMO use (≈64% to 38%) and decreased chronic lung disease; mortality similar between groups; no reported increase in renal dysfunction.
Konduri et al., 2004 [108]	Term/near-term neonates with early hypoxic respiratory failure (early vs conventional iNO initiation)	299	Early group: iNO started at OI 15–25; conventional group: initiated at OI ≥25; initial dose 20 ppm with stepwise weaning	Death or ECMO	Early iNO produced faster improvement in oxygenation but did not significantly reduce death or ECMO; ventilation duration modestly reduced; no apparent increase in acute kidney injury (AKI).
Barrington & Finer, Cochrane review, 2017 (term/near-term) [109]	Term/near-term infants with hypoxic respiratory failure/PPHN; multiple RCTs	~1300	Most trials used 5–20 (up to 80) ppm as rescue therapy initiated in moderate–severe hypoxemia	Death, ECMO, chronic lung disease, long-term neurodevelopment	iNO reduced the composite of death or ECMO, driven by lower ECMO use, but did not improve mortality alone, bronchopulmonary dysplasia, or long-term neurodevelopment; no consistent signal of nephrotoxicity.
Taylor et al., 2004 [110]	Adults with acute lung injury/ARDS without pre-existing multi-organ failure	385	Fixed-dose 5 ppm, continued up to day 28, extubation, or death	Ventilator-free days at day 28	iNO transiently improved PaO_2_/FiO_2_ but did not increase ventilator-free days or reduce mortality; no strong renal signal within this single trial, but subsequent pooled analyses link ARDS cohorts to higher AKI risk.
Gebistorf et al., Cochrane review, 2016 (ARDS) [80]	Pediatric and adult ARDS of various etiologies; 14 RCTs	~1300	Typically 5–20 ppm as short-term rescue, sometimes titrated and continued for several days	Mortality (28/90-day), ventilator-free days, oxygenation, renal outcomes	iNO consistently improved short-term oxygenation but did not reduce mortality, ventilator-free days, or ICU/hospital length of stay; meta-analysis showed increased risk of renal dysfunction or AKI, especially in high-dose/severe ARDS subgroups.
Ruan et al., meta-analysis, 2015 (renal outcomes) [82]	Mixed populations (predominantly ARDS and cardiac/transplant surgery)	17 RCTs, 1280 pts	Doses 5–80 ppm; timing varied according to indication	Incidence of renal dysfunction/AKI	Overall, iNO use was associated with increased risk of renal dysfunction (RR ≈ 1.4), largely driven by ARDS/critically ill cohorts; in cardiac surgery/CPB trials, no consistent harmful renal signal was observed.
Miller et al., 2000 [15]	High-risk infants and young children after congenital heart surgery with CPB, prone to postoperative pulmonary hypertensive crises	124	Prophylactic 10 ppm started before separation from CPB and continued in the early postoperative period	Incidence of pulmonary hypertensive crises; time to meet extubation criteria	iNO significantly reduced pulmonary hypertensive crises and shortened time to extubation readiness; no increase in AKI or other major adverse effects reported.
Schlapbach et al. (NITRIC trial), 2022 [98]	Children < 2 years undergoing complex congenital heart surgery with CPB (multicenter RCT)	1371	~20 ppm delivered via the CPB oxygenator from initiation of bypass until separation; some centers continued low-dose iNO postoperatively	Ventilator-free days at day 28	No significant differences in ventilator-free days, 28-day mortality, low cardiac output syndrome, or major postoperative complications; prespecified renal outcomes showed no clear difference between groups.
Lei et al., 2018 [111]	Adults undergoing multivalve cardiac surgery with prolonged CPB, at high risk of CSA-AKI	244	80 ppm via the CPB oxygenator during bypass, followed by lower-dose iNO inhalation for 24 h postoperatively	Postoperative AKI (KDIGO); CKD progression and MAKE at 90 days/1 year	iNO reduced AKI incidence (≈64% to 50%) and decreased progression to stage 3 CKD and major adverse kidney events at 90 days and 1 year; no increase in hypotension or bleeding, suggesting a renal-protective effect in this high-risk population.
Kamenshchikov et al., 2022 [112]	Adults undergoing valve and other cardiac procedures with CPB	77	NO (≈40–80 ppm) delivered via the CPB oxygenator from onset of bypass until separation; no routine postoperative inhalation	Postoperative AKI (KDIGO)	iNO reduced overall AKI incidence (≈42% to ≈21%) and lowered severe AKI and need for renal replacement therapy; no major safety concerns, supporting early intra-CPB NO for CSA-AKI prevention.
Hu et al., meta-analysis, 2019 (CPB-AKI) [113]	Adult cardiac surgery patients undergoing CPB	5 RCTs, 579 pts	10–80 ppm, mainly administered during CPB; some trials extended treatment postoperatively	AKI incidence, ICU/hospital length of stay, bleeding, methemoglobinemia	Pooled analysis showed that intra-CPB NO reduced AKI risk, particularly when started at the beginning of CPB; no significant effect on ICU/hospital stay or bleeding; methemoglobin levels rose slightly but without major clinical consequences.
Abouzid et al., meta-analysis, 2023 (CPB iNO) [97]	Pediatric and adult patients undergoing CPB cardiac surgery	17 RCTs, 2897 pts	Mostly 10–40 ppm during CPB (some trials used 80 ppm); some continued iNO postoperatively	Mortality, mechanical ventilation duration, ICU/hospital stay, CPB time	Overall mortality and CPB time were similar between groups; iNO modestly shortened ICU stay and significantly reduced ventilation duration in pediatric subgroups; across included trials there was no consistent nephrotoxic signal, and findings from high-risk adult cohorts (e.g., Lei, Kamenshchikov) suggest possible renal protection.
Arora et al., 2025 [114]	Adults with pre-existing endothelial dysfunction undergoing prolonged CPB (>90 min) (double-blind, single-center placebo-controlled RCT)	250	NO 80 ppm delivered via CPB oxygenator during bypass, continued postoperatively via ventilator/facemask for 24 h vs placebo (nitrogen–oxygen mixture)	Postoperative AKI incidence (KDIGO)	AKI incidence was similar between groups (NO 44.0% vs control 43.2%; adjusted OR ~1.00); AKI severity and renal replacement therapy at all time points were also similar, not supporting routine perioperative NO for AKI prevention in this high-risk population.
Di Fenza et al. (Nitric Oxide Investigators), 2023 [83]	Mechanically ventilated adults with COVID-19 acute hypoxemic respiratory failure (multicenter phase II RCT)	193	High-dose iNO 80 ppm for 48 h vs usual care (no placebo); initiated while mechanically ventilated	Change in PaO_2_/FiO_2_ at 48 h	High-dose iNO improved PaO_2_/FiO_2_ at 48 h and increased the proportion achieving PaO_2_/FiO_2_ ≥ 300 mmHg by day 28, but duration of ventilation and 28-/90-day mortality were similar; no serious safety signals reported.
